# Infant hyperglycemic excursions by saliva 1,5-anhydroglucitol varied by weight status, rapid weight gain, and mode of feeding: an exploratory secondary analysis

**DOI:** 10.1016/j.ajcnut.2026.101254

**Published:** 2026-02-26

**Authors:** Douglas Taren, Kinzie Matzeller, Stephanie P Gilley, Mikayla Hernandez, Jennifer F Kemp, Michael Armstrong, Katrina Doenges, Nancy F Krebs, Minghua Tang

**Affiliations:** 1 Department of Pediatrics, Section of Nutrition, University of Colorado Anschutz Medical Campus, Aurora, CO, United States; 2 Department of Food Science and Human Nutrition, Colorado State University, Fort Collins, CO, United States; 3 Skaggs School of Pharmacy and Pharmaceutical Sciences, University of Colorado Anschutz Medical Campus, Aurora, CO, United States

**Keywords:** 1,5-anhydroglucitol, anthropometry, hyperglycemic excursions, infants, rapid weight gain

## Abstract

**Background:**

Early infant feeding and weight gain can impact the development of chronic diseases. 1,5 anhydroglucitol (1,5-AG) is a measure of hyperglycemic excursions (HEs) and decreases during HEs due to increased urinary excretion.

**Objectives:**

We hypothesized that HEs as measured by 1,5-AG are associated with infant rapid weight gain (RWG).

**Methods:**

Infants from the Maternal and Infant Nutrition Trial, a randomized controlled feeding trial, participated in this exploratory secondary analysis. Saliva from 127 infants aged 5 mo was collected before the introduction of complementary foods. Saliva 1,5-AG concentration was measured using liquid chromatography-mass spectrometry and log transformed for statistical analyses. Demographic, anthropometric, and feeding mode [exclusively human milk-fed (EHMF), exclusively formula fed (EFF), and mixed fed (MF)] data were collected. RWG was classified as an increase in the weight-for-age *z*-score (WAZ) >0.67 from birth to 5 mo.

**Results:**

Saliva 1,5-AG concentrations were negatively correlated with an increase of WAZ at 5 mo (*r* = −0.238, *P* = 0.01). EHMF and MF infants had significantly greater log transformed 1,5-AG μg/dl values (−0.252 ± 0.639 and 0.332 ± 0.912, respectively) than EFF infants (−1.754 ± 0.705, *P* = 0.001). Birth weight, but not sex, age, or maternal body mass index, was positively associated with WAZ changes. Saliva 1,5-AG concentrations remained inversely associated with the change in WAZ values in adjusted regression models when controlling for birth weight (β: −0.214; 95% confidence interval: −0.38, −0.05), but not when mode of feeding was included. The odds ratio for 1,5-AG (0.50; 95% confidence interval: 0.28, 0.90) was also significantly associated with RWG using logistic regression controlling for birth weight.

**Conclusions:**

These results suggest that human milk-fed infants have fewer HEs than EFF infants. Lower saliva 1,5-AG concentrations were associated with an increase in WAZ and RWG. Additional analyses are needed to evaluate the underlying drivers of HEs and their association with infant growth and health.

This study was registered at clinicaltrials.gov as NCT05012930.

## Introduction

Growth trajectories during infancy have been shown to strongly correlate with obesity risks and metabolic health later in life [[1], [2], [3]]. Parameters describing infant growth status include weight-for-age *z*-score (WAZ), which use WHO standards that are age- and sex-adjusted, and rapid weight gain (RWG), defined as WAZ change >0.67 [4]. RWG or excessive weight gain proportionate to length gain, during infancy is one of the main risks for the development of obesity and metabolic syndrome in children and adolescents [5,6]. Recently it was reported that infants who were formula fed and those with RWG were at greater risk of developing obesity by age 6 y [7]. Children and adolescents with obesity are much more likely to have obesity as adults and could therefore experience an earlier onset of metabolic syndrome [[8], [9], [10]]. Unfortunately, once established, obesity and its comorbidities can be difficult to treat. Thus, preventing the development of obesity and metabolic syndrome early in life is critical for improving public health and reducing the burdens associated with obesity-related diseases. To achieve such goals and identify infants at risk, noninvasive measures of metabolic health indicators need to be implemented. More importantly, the relationship between parameters of growth (e.g., RWG and WAZ) and infant metabolic health is not clearly understood, partially due to the lack of sensitive assessments of metabolic function for healthy infants participating in growth studies.

Metabolic health parameters, such as glycemic control, are not easily accessible in infants due to the feasibility of blood sample collections [11]. In recent years, 1,5-anhydroglucitol (1,5-AG), a measure of hyperglycemic excursions (HEs) in the preceding 10–14 d, is emerging as a validated biomarker of glycemic control that can be measured noninvasively in saliva [12,13]. 1,5-AG concentration decreases during HEs due to increased urinary excretion, and 1,5-AG concentration is lower in patients with diabetes and cardiovascular disease than in healthy adult controls [14]. Serum 1,5-AG concentrations have also been shown to be negatively related to the mean postprandial glucose concentration using continuous glucose monitoring [15]. Validation studies have concluded that saliva 1,5-AG concentrations can accurately reflect serum 1,5-AG concentrations and can be used to assess short-term glycemic control noninvasively [16]. The primary outcome of the present analysis is the evaluation of infant HEs, measured by saliva 1,5-AG concentration, and their associations with growth as measured by changes in WAZ and the risk of RWG in United States infants who received human milk and/or infant formula. We hypothesized that HEs are associated with greater obesity risks; thus, saliva 1,5-AG concentration is negatively associated with RWG and WAZ. The secondary outcome of this analysis is to test the hypothesis that exclusively human milk-fed (EHMF) infants would have better glycemic control than infants receiving formula, as reflected by higher saliva 1,5-AG concentrations [7], using data collected during the Maternal and Infant Nutrition Trial (MINT), a randomized controlled feeding trial [17].

## Methods

### Subjects

Saliva was collected from 127 healthy infants aged 5 mo from the Denver, Colorado area who were enrolled in a randomized trial (MINT) on the impact of complementary foods on infant growth and the gut microbiome, the intervention of which was conducted between 2021 and 2025, with ongoing follow-up of participants [17]. The sample for saliva collection included all participants who enrolled after this procedure was implemented and for whom biomarker data were available. Participants’ saliva was collected at baseline of the trial, prior to the introduction of solid foods, and before the study intervention was initiated. Inclusion criteria in the parent trial included the following: *1*) singleton birth; *2*) full-term (≥37 weeks of gestation); *3*) no exposure to antibiotics; *4*) no exposure to complementary foods; and *5*) generally healthy without conditions affecting nutrition or growth.

Written informed consent was obtained from the infant’s legal guardian. The study was approved by the Colorado Multiple Institutional Review Board (Protocol 20-2232) and is registered at clinicaltrials.gov as identifier NCT05012930. The data used from this study include demographic information, infant and parental health, maternal and infant anthropometric measures, mode of feeding, and the analysis of infant saliva for its 1,5-AG concentration.

### Data collection

Infant caregivers were given a questionnaire at the baseline visit to report the infant and parental ethnicity (Hispanic compared with non-Hispanic), race (Asian, Black/African American, White, Other, or More than one race), sex, and socioeconomic status. An interview was conducted to obtain information about the mode of delivery and infant birth weight and length. Family history regarding the presence of chronic diseases including obesity, cardiovascular disease, type 2 diabetes mellitus, and allergies were also collected. Infant feeding habits for the previous month were assessed and classified as EHMF and exclusively formula fed (EFF). Children who received mixed human milk and formula feeding (MF) were included to determine if these children would have a response between those of EHMF and EFF infants. The formulas were put into the following groups: Standard, Modified (partially hydrolyzed with or without decreased lactose), and Other (Extensively hydrolyzed, Soy and Goat Milk Formula).

Infant weight (grams) and length (centimeters) were measured in triplicate at 5 mo by trained pediatric research nurses at Children’s Hospital Colorado. The mean values were used to generate *z*-scores for WAZ, length-for-age (LAZ), and weight-for-length (WLZ) based on the WHO Growth Standards for 0 to 24 mo [18]. The birth lengths used in this report were from the measurements taken at newborn nurseries before infants were enrolled in the study. Given that these measures were not taken using a research protocol, we report birth lengths for descriptive purposes only. Thus, we have not studied how 1,5-AG is associated with LAZ and WLZ values. Additionally, mothers self-reported their height and weight at the baseline visit.

Mothers were instructed to have their infants be fasted for 2 h before saliva samples were collected. At a minimum, samples were collected ≥30 min to 1 h after any feeding and during daylight hours. Saliva was collected at a home visit with a SalivaBio Infant Swab (Salimetrics) collection kit. The swab was held in the infant’s mouth for 60–90 s, and then placed in a centrifuge tube on ice for transport to the Pediatric Nutrition Laboratory. The tube was centrifuged at 1500 × *g* for 15 min (4 °C), and saliva was transferred to a cryovial tube for storage at −80 °C. The dates when the samples were collected and analyzed were recorded to determine duration of storage.

Saliva samples were analyzed for 1,5-AG as previously described using liquid chromatography-mass spectrometry (LC/MS) with minor modifications [19]. 1,5-AG analyses were performed with a 1260 HPLC system (Agilent) using an Agilent 6490 mass spectrometric instrument with a triple quadrupole mass spectrometer in negative ionization mode. The lower limit of quantitation for 1,5-AG was 0.0325 μg/dL and the upper limit of quantitation was 50 μg/mL for both saliva and plasma. When sample values were outside the upper limit of quantitation, samples were diluted and reanalyzed. The recovery of 1,5-AG spiked at concentrations from 1 μg/mL to ≤50 μg/mL was 98.3% ± 1.8% for saliva and 108.5% ± 8.9% for plasma. The correlation between 93 paired log-transformed serum and saliva 1,5-AG samples had an *r* = 0.814 (*P* < 0.001). When stored over 9 mo at −20 °C, the mean (±1 SD) coefficient of variation was 5.75 ± 3.38.

### Data analysis

All statistical analyses were conducted using STATA 18.5. Initial descriptive statistics were performed to determine distributions and to identify potential outliers (none identified) using scatter plots and frequency tables (Figure 1). The means, SDs, and frequency are presented. Analyses were conducted using all available data; case-wise deletion was applied for missing descriptive data. Inferential statistics used the log-transformed values for 1,5-AG as the raw values were not normally distributed [20,21]. Correlations and simple regressions were calculated for the association between the saliva 1,5-AG concentrations and total weight gain from birth to 5 mo, mean grams gained per day, and change in the WAZ. Chi-square analysis was used to determine significant differences for categorical data. Student *t*-test was used to determine if the mean 1,5-AG concentrations were different between infants with and without RWG as defined as ≥0.67 SD increase in WAZ between birth and 4 to 6 mo [4,22]. Analysis of variance (ANOVA) was used to determine differences in the saliva 1,5-AG concentration between feeding method groups and Student *t*-tests for specific differences between the EFF and EHMF groups. Comparisons with the MF group were not done due to the heterogeneity of formulas in the group. Similarly, we compared the 1,5-AG values for infants who were fed different formula types, and the ‘Other’ category was not included in the individual comparisons due to the heterogeneity of formulas within that group as it included hydrolyzed formulas and goat’s milk formulas. Correlations were also calculated to assess for potential confounders with the saliva 1,5-AG concentrations and with weight gain. These confounders were then included in multiple regression and multiple logistic regression models to determine if they modified the associations between the saliva 1,5-AG concentrations and the weight gain measures. The covariates placed in a full regression models included feeding method (EHMF, EFF, or MF), birth weight (grams), age in days at sample collection, infant sex (male or female) and maternal BMI (in kg/m^2^). Reduced models were then computed based on the factors that were significant in the full models.

**FIGURE 1:**
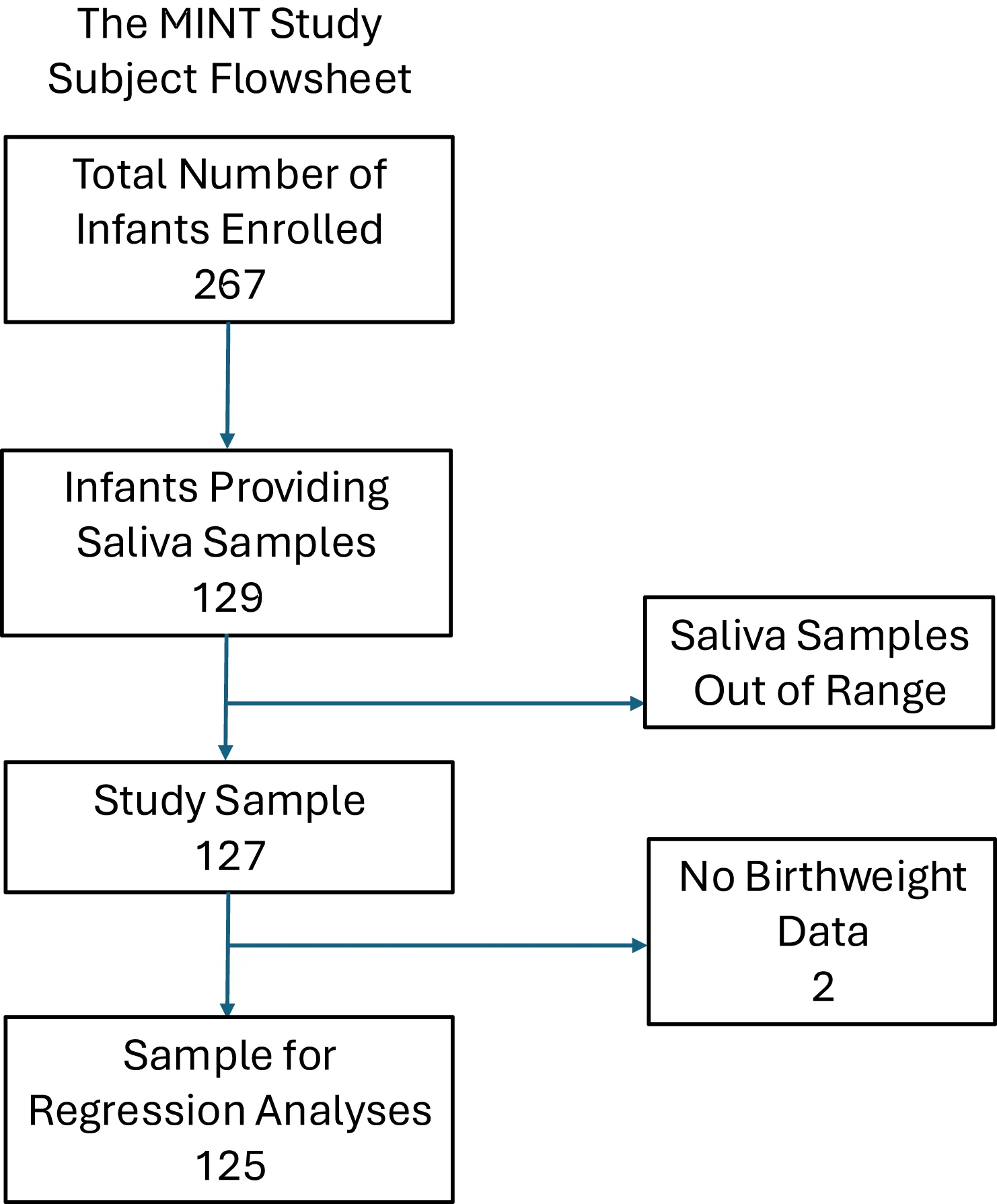
Participant flowchart. MINT, Maternal and Infant Nutrition Trial.

## Results

### Subjects

One hundred twenty-seven infants were included in the current analyses; a flowchart of the selection of participants for this analysis can be found in Figure 1. The mean (± SD) age of the infants was 163.7 ± 16.1 d, and 48.0% were female (Table 1). Their mean gestational age at birth was 39.0 ± 1.3 wk. The majority (59.8%) of infants were classified as EHMF, 20.5% were EFF, and 19.7% were in the MF group. Most of the infants were from families that had an annual income >$100,000 (68.9%) (Table 1), and 72.4% self-identified as White (Hispanic and non-Hispanic data not shown). There were no significant differences between this subsample compared with the rest of the sample from the MINT study for age, sex, gestational age, mode of feeding, and income.

**TABLE 1:** Descriptive subject population characteristics and anthropometrics

Sample characteristics	Value
Age (d)	163.7% ± 16.1
Female	48%
Race	
Asian	3.9%
Black/African American	1.6%
White	74.0%
Other	3.1%
More than one race	17.3%
Income (annual, *n* = 122)	
<$50,000	7.4%
$50,000-$100,000	23.8%
$100,00-$150,000	23.0%
>$150,000	45.9%
Feeding method	
Exclusively human milk	59.8%
Exclusively formula	20.5%
Mixed	19.7%
Birth measures	
Birth weight (g) (*n* = 125)	3278 ± 456
Birth length (cm) (*n* = 119)	51 ± 3
Birth WAZ (*n* = 125)	−0.07 ± 0.85
Current measures (5 mo)	
Weight (g)	6891.2 ± 896.4
WAZ	−0.51 ± 1.00
LAZ	−0.78 ± 0.93
WLZ	0.03 ± 1.02
Change in weight from birth	
Total weight gained (g)	3593.1 ± 0.8
Weight gain per day (g/d)	22.1 ± 4.9
Change in WAZ	−0.45 ± 1.11
Maternal anthropometric data	
Maternal weight (kg)	72.4 ± 15.6
Maternal height (cm)	165.4 ± 7.1
Maternal BMI (kg/m^2^)	26.4 ± 5.2

Data are presented as mean ± standard deviation or percentage. *n* =127 unless otherwise stated. Age, sex, race, and feeding method represent infant characteristics. Income represents gross family income.

Abbreviations: BMI, body mass index; LAZ, length-for-age z-score; WAZ, weight-for-age z-score; WLZ, weight-for-length z-score.

### Anthropometry

The mean (±1 SD) maternal height and weight were 165.4 ± 7.1 cm and 72.4 ± 15.6 kg. The mean maternal BMI was 26.4 ± 5.2. The mean birth weight and birth WAZ were 3278 ± 456 g and −0.07 ± 0.95, respectively (Table 1). The mean weight at the time of the study visit (∼5 mo) was 6891 ± 896 g, and the mean weight gain since birth was 22.1 ± 5 g/d. The mean change in the WAZ was −0.45 ± 1.11. RWG was present in 15.7% (*n* = 20) of the infants. There were no significant differences between this subsample compared with the rest of the sample from the MINT study for the anthropometric measurements.

### 1,5-AG concentrations

The mean saliva 1,5-AG concentration was 0.9808 ± 0.9815 μg/dL, and the values were log transformed for analysis. The saliva 1,5-AG concentrations for EHMF infants (1.22 ± 1.03 μg/dL) and MF infants (1.03 ± 0.94 μg/dL) were significantly greater (*P* ≤ 0.001) than those for EFF infants (0.22 ± 0.18 μg/mL). Mean saliva 1,5-AG values can be found in Table 2. Infant birth and maternal characteristics were not associated with the saliva 1,5-AG values (Table 2). Additionally, the 1,5-AG concentrations were significantly different (*P* ≤ 0.05) between formula groups (Table 3). The 1,5-AG concentration for infants fed Modified formula (1.15 ± 1.34 μg/dL) and the Other formula group (0.72 ± 0.61 μg/dL) were significantly greater (*P* ≤ 0.05) than those for infants fed Standard formula (0.43 ± 0.57 μg/dL), with no difference between the Modified and Other formula fed infants (Table 3).

**TABLE 2:** 1,5-Anhydroglucitol (1,5-AG) concentrations by feeding method and correlations with birth measures and maternal anthropometric measures

Mean 1,5-AG concentrations		
	Mean ± SD	
Saliva 1,5-AG (μg/dL)	0.98 ± 0.98	
Log Saliva 1,5-AG	−0.440 ± 0.981	

Feeding method		

	Saliva 1,5-AG concentration μg/dL Mean ± 1SE	Log-transformed 1,5-AG concentration^1^ Mean ± 1SE
EHMF	1.22 ± 1.03	−0.252 ± 0.639
EFF	0.22 ± 0.18	−1.754 ± 0.705
Mixed	1.03 ± 0.94	−0.332 ± 0.912

Correlation coefficients with birth characteristics		

	Saliva 1,5-AG concentration (μg/dL)	Log-transformed 1,5-AG concentration (μg/dL)
Gestational age	0.010	0.034 *P* = 0.70
WAZ (*n* = 125)	0.151	0.095 *P* = 0.29
Birth weight (*n* = 125)	0.136	0.092 *P* = 0.30

Correlation coefficients with maternal characteristics		

	Saliva 1,5-AG concentration (μg/dL)	Log-transformed 1,5-AG concentration (μg/dL)
Mother’s weight	−0.090	−0.034 *P* = 0.71
Mother’s height	0.004	0.092 *P* = 0.031
Mother’s BMI	−0.096	−0.063 *P* = 0.48

*n* = 127 unless specified in table. Values presented as mean ± SD or Pearson correlation coefficient. Statistical tests performed with log-transformed 1,5-AG concentrations. ANOVA used for difference between feeding method and Student’s *t*-test for comparing the EHMF and EFF groups. Comparisons with the mixed fed group were not done due to the heterogeneity of formulas in the group.

Abbreviations: ANOVA, analysis of variance; BMI, body mass index; EFF, exclusively formula fed; EHMF, exclusively human milk-fed; Mixed, human milk-fed and formula fed; SD, standard deviation; SE, standard error.

1 ANOVA, *P* = 0.0001; EHMF vs. EFF, *t*-test, *P* = 0.0001.

**TABLE 3:** 1,5-AG values by type of formula

Type of formula (*n* = 51)	1,5-AG concentration, μg/dL (mean ± SD)	Log 1,5-AG concentration (mean ± SD)	*P*^1^
Standard (*n* = 31)^2^	0.43 ± 0.57	−1.39 ± 1.02	0.02
Modified (*n* = 9)	1.14 ± 1.34	−0.45 ± 1.14	
Other (*n* = 11)	0.72 ± 0.51	−0.63 ± 0.89	

Statistical tests performed with log-transformed 1,5-AG concentrations. The ‘Other’ category was not included in the individual comparisons due to the heterogeneity of formulas within that group as it included hydrolyzed formula (*n* = 4), soy-based formula (*n* = 1), goat’s milk formula (*n* = 2), and multiple formulas used (*n* = 4).

Abbreviations: 1,5-AG, 1,5-anhydroglucitol; ANOVA, analysis of variance; SD, standard deviation.

1 ANOVA used to determine *P* values between the 3 groups.

2 Standard and Gentle Formulas were compared with *t*-test with *P* = 0.02.

### Weight gain

The log saliva 1,5-AG concentrations (Table 4) were significantly correlated with total weight gain (*r* = −0.202; *P* ≤ 0.05), weight gain as grams per day (*r* = −0.191; *P* ≤ 0.05) and change in the WAZ (*r* = −0.238; *P* ≤ 0.01). Birth weight was not significantly correlated with total weight gain (*r* = −0.126) or the mean grams of weight gain per day (*r* = 0.083), but it was significantly correlated (*r* = −0.522, *P* ≤ 0.001) with the change in the WAZ from birth. The mean ± 1 SD for total weight gain was greater (*P* ≤ 0.05) for males (3833.0 ± 91.1 g) than for females (3341.0 ± 104.4 g). Similarly, the weight gain as grams per day was also greater (*P* ≤ 0.01) for males (23.2 ± 0.5 g/d) than for females (20.9 ± 0.7 g/d). However, the mean change in the WAZ was not significantly different between males (−0.50 ± 0.13) and females (−0.39 ± 0.15) because anthropometric *z*-scores are sex-specific. Age was also correlated with total weight gain (*r* = −0.239, *P* ≤ 0.01), weight gain as grams per day (*r* = −0.191, *P* ≤ 0.05), but not with the change in the WAZ from birth (*r* = −0.015). Additionally, the correlations were significant (Table 4) between the log-transformed 1,5-AG concentrations and total weight gain (*r* = −0.202, *P* ≤ 0.05), weight gain grams per day (*r* = −0.191, *P* ≤ 0.05), and change in WAZ from birth (*r* = 0.238, *P* ≤ 0.01).

**TABLE 4:** Saliva 1,5-AG correlations with changes in weight measures and age

Correlation coefficients with saliva 1,5-AG Concentrations (*n* = 125)			*P*
	Saliva 1,5-AG concentration (μg/dL)	Log-transformed 1,5-AG concentration (μg/dL)	
Total weight gain (g)	−0.153	−0.202	0.02
Weight gain (g/d)	−0.164	−0.191	0.03
WAZ change from birth to 5 mo	−0.186	−0.238	0.01
Correlation coefficients with age			
Total weight gain (g)	−0.239		0.01
Weight gain (g/d)	−0.211		0.02
WAZ change from birth to 5 mo	−0.015		0.87

Statistical tests performed with log-transformed 1,5-AG concentrations. Pearson correlation coefficients are shown.

Abbreviations: 1,5-AG, 1,5-anhydroglucitol; WAZ, weight-for-age z-score.

### Rapid weight gain

Log saliva 1,5-AG concentrations were significantly lower (*P* < 0.05) for the infants who had RWG (0.64 ± 0.12 μg/mL) than for those who did not (1.04 ± 0.1 μg/mL). The percentage of participants with RWG was not significantly different between males (15.2%) and females (16.4%). The percentage of EFF infants with RWG (26.9%) was greater than infants in the EHMF group (14.5%) and the MF group (8.0%), but these differences were not significantly different with a chi-square test (Table 5).

**TABLE 5:** Rapid weight gain with saliva 1,5-AG concentrations, age, feeding method and sex

	Rapid weight gain		*P*
	No (*n* = 107) Mean ± 1SE	Yes (*n* = 20) Mean ± 1SE	
Saliva 1,5-AG (μg/dL)	1.04 ± 0.10	0.64 ± 0.12	N/A
Log Saliva 1,5-AG	−0.35 ± 0.09	−0.89 ± 0.24	0.02
Age (d)	164.1 ± 1.6	161.7 ± 3.7	0.54
Feeding method	Rapid weight gain (%)		
EHMF	14.5		0.16
EFF	26.9		
Mixed	8.0		
Sex			
Male	15.5		0.85
Female	16.4		

Rapid weight gain was defined as a change in weight-for-age *z*-score ≥0.67 from birth to 5 mo. Means were compared with *t*-tests. Frequencies were compared with chi-square tests.

Abbreviations: 1,5-AG, 1,5-anhydroglucitol; EFF, exclusively formula fed; EHMF, exclusively human milk-fed; Mixed, human milk-fed and formula-fed; N/A, not applicable; SE, standard error.

### Regression models

#### Total weight gain

Factors associated with the total weight gain in a full regression model included age and sex, whereas mode of feeding and birth weight were not associated. Given the high association between mode of feeding and the saliva 1,5-AG concentrations, a reduced model without mode of feeding indicated that the saliva 1,5-AG concentrations (*P* = 0.06), age (*P* = 0.025) and sex (*P* = 0.002) were associated with total weight gain whereas birth weight was not (Table 6).

**TABLE 6:** Regression models associations with infant weight measures

	Β	95% CI	*P*
**Weight gain (g)**			
Full model			
Log Saliva 1,5-AG	−57.42	−247.44, 132.60	0.551
EFF (ref EHMF)	258.8	−218.88, 736.57	0.285
Mixed (ref EHMF)	141.0	−211.72, 493.84	0.430
Maternal BMI	6.02	−20.76, 32.80	0.657
Birth weight (g)	−0.174	−0.47, 0.12	0.248
Age (d)	10.01	1.43, 18.59	0.023
Female (ref male)	−419.7	−694.89, −144.58	0.003
Reduced model			
Log Saliva 1,5-AG	−129.5	−265.89, 6.90	0.063
Birth weight (g)	−0.165	−0.46, 0.13	0.271
Age (d)	9.56	1.20, 17.92	0.025
Female	−433.74	−704.51, 162.97	0.002
**Weight gained per day (g/d)**			
Full model			
Log Saliva 1,5-AG	−0.362	−1.52, 0.80	0.538
EFF (ref EHMF)	1.785	−1.13, 4.70	0.228
Mixed (ref EHMF)	0.916	−1.23, 3.07	0.401
Maternal BMI	0.040	−0.12, 0.20	0.630
Birth weight (g)	−0.0009	−0.003, 0.001	0.302
Age (days)	−0.075	−0.13, −0.02	0.005
Female (ref male)	−2.451	−4.13, −0.77	0.005
Reduced model			
Log Saliva 1,5-AG	−0.859	−1.69, −0.02	0.043
Birth weight (g)	−0.0009	−0.003, 0.001	0.331
Age (d)	−0.078	−0.13, −0.03	0.003
Sex	−2.548	−4.20, −0.89	0.003
**Weight-for-age *z*-score**			
Full model			
Log Saliva 1,5-AG	−0.081	−0.32, 0.15	0.494
EFF (ref EHMF)	0.480	−0.11, 1.07	0.110
Mixed (ref EHMF)	0.157	−0.28, 0.59	0.476
Maternal BMI	0.003	−0.03 0.04	0.857
Birth weight (g)	−0.0012	−0.002, −0.001	0.000
Age (d)	−0.0003	−0.01, 0.01	0.961
Reduced model			
Log Saliva 1,5-AG	−0.2145	−0.38, −0.05	0.013
Birth weight (g)	−0.0012	−0.002, −0.001	0.000

**Logistic rapid weight gain**			

	**Odds ratio**	**95% CI**	***P***

Full model			
Log Saliva 1,5-AG	0.433	0.164, 1.14	0.091
EFF (ref EHMF)	0.802	0.103, 6.218	0.833
Mixed (ref EHMF)	0.603	0.104, 3.505	0.573
Maternal BMI	1.036	0.928, 1.16	0.526
Birth weight (g)	0.997	0.996, 0.999	0.001
Age (d)	0.98	0.946, 1.016	0.266
Reduced model			
Log Saliva 1,5-AG	0.50	0.28, 0.90	0.020
Birth weight (g)	0.997	0.996, 0.999	0.001

*n* = 127. Rapid weight gain is defined as a change in WAZ ≥0.67 from birth to 5 mo. β estimates were calculated using multiple regression; odds ratios were calculated from multiple logistic regression modeling. Full model includes log saliva 1,5-AG, mode of feeding, maternal BMI, birth weight (grams), and age (days); reduced model includes only log saliva 1,5-AG and birth weight, which were significant in the full model.

Abbreviations: 1,5-AG, 1,5-anhydroglucitol; BMI, body mass index; CI, confidence interval; EFF, exclusively formula fed; EHMF, exclusively human milk-fed.

#### Weight gain as grams per day

Age and sex were associated with the mean weight gain in grams per day in a full regression model that also included saliva 1,5-AG concentrations, mode of feeding, birth weight, and maternal BMI. However, a reduced model that did not include mode of feeding resulted in a significant association of saliva 1,5-AG concentrations [β: −0.859; 95% confidence interval (CI): −1.69, −0.02; *P* = 0.043], sex (β: −2.548; 95% CI: −4.20, −0.89; *P* = 0.003), and age (β: −0.078; 95% CI: −0.13, −0.03; *P* = 0.003) with weight gain per day; birth weight was not significantly associated (Table 6).

#### WAZ change and RWG

The full regression model to test for associations between saliva 1,5-AG concentration and change in WAZ from birth to study enrollment at 5 mo included feeding method, maternal BMI, birth weight, and age (Table 6). Of these, only birth weight was significantly associated with the change in WAZ. In a reduced model that excluded mode of feeding and nonsignificant factors, there was a significant association between WAZ change and both saliva 1,5-AG concentration (β: −0.2145; 95% CI: −0.38, −0.01; *P* = 0.013) and birth weight (β: −0.0012; 95% CI: −002, −001; *P* = 0.001) (Table 6). Infants with higher 1,5-AG concentration (Table 6) had lower odd ratios (ORs) of RWG (OR: 0.503; 95%CI: 0.28, 0.89; *P* = 0.02). Higher birth weight was also associated with lower odds of RWG (OR: 0.997; 95%CI: 0.996, 0.999; *P* = 0.001).

## Discussion

In this analysis, we found that saliva 1,5-AG concentrations were significantly lower in infants with RWG between birth and 5 mo than in those without. Additionally, 1,5-AG concentration was significantly lower in EFF infants than EHMF or MF infants. To our knowledge, this is the first study that has analyzed the relationship between RWG in infants and 1,5-AG concentration, finding that lower salivary 1,5-AG values at 5 mo were associated with total weight gain, rate of weight gain, and higher odds of RWG between birth and 5 mo. Additionally, 1,5-AG concentration was lower in infants with RWG (0.64 ± 0.12 μg/mL) than in infants without (1.05 ± 0.1 μg/mL), which suggests a relationship between weight gain and HEs represented by 1,5-AG. These findings suggest that saliva 1,5-AG may serve as a feasible, noninvasive screening tool for early identification of infants at risk of altered growth trajectories.

Low birth weight has previously been associated with a higher risk of RWG, which may be attributed to catch-up growth and recommendations from pediatricians; conversely, children with higher birth weights are less likely to experience RWG and may show “catch down” growth [23,24]. Therefore, the finding that a greater birth weight was associated with lower odds of RWG supported having it as an important covariate in our models. Interestingly, although the prevalence of RWG was greater in children who were EFF, there was no statistically significant difference between feeding groups. Having only 20 children with RWG may have limited our power to detect differences.

In adults, lower 1,5-AG values have also been associated with higher BMI [25]. In children with type 1 diabetes, 1,5-AG was associated with lower BMI and adiposity [26]. These findings and ours suggest there may be a relationship between HEs and body size and body composition during infancy and childhood.

Because the concentration of 1,5-AG differs between human milk (∼11.7 μg/mL at 1-mo postpartum) and infant formula (<0.5 μg/mL) [27], we tested for differences in saliva 1,5-AG by primary feeding mode. In our study, salivary 1,5-AG concentration was consistently lower in EFF infants than in EHMF and MF infants, with high correlation between mode of feeding and saliva 1,5-AG concentration. As EFF infants had lower 1,5-AG values, they may have experienced more HEs than EHMF and MF infants [28], potentially related to formula additives and other composition differences between formula and human milk. For example, the absorption and bioavailability of sugars in infant formulas may be associated with more HEs compared with human milk. In our analysis, infants receiving partially hydrolyzed formulas with or without reduced lactose had 1,5-AG concentrations closer to those of EHMF infants, whereas those receiving Standard formula had the lowest 1,5-AG concentrations, suggestive of more HEs [29]. This difference is potentially important for future diabetes risk as 1,5-AG concentrations are correlated with higher insulin resistance, lower insulin secretion, and decreased glucose control [30]. These findings suggest the type of formula, and the specific carbohydrates in infant formula, may affect HEs and 1,5-AG values and warrant more research.

Although our findings may be related to the composition of the feeds, it could also be related to feeding behaviors. Prior work by others showed that weight gain during infancy may be connected to the act of bottle feeding, rather than whether the bottle contained formula or expressed human milk [31,32]. In addition, human milk has been associated with greater infant satiety responsiveness and decreased overfeeding [33,34]. Little is known about the relationship between infant carbohydrate metabolism and feeding behaviors. Future studies should be performed to test whether saliva 1,5-AG concentration differs based on feeding behaviors and/or total calorie consumption, or if differences relate to feeding composition.

Dietary sources for 1,5-AG include dairy and soy but appear to have minimal impacts on 1,5-AG concentrations [35,36]. Adults who reported regularly consuming cow milk had lower serum 1,5-AG concentrations than minimal milk drinkers [37]. Additionally, the serum 1,5-AG concentration was not associated with soybean intake [[37], [38], [39]]. Similarly, infants fed cow milk- or soy-based formulas had lower urinary 1,5-AG concentrations than human milk-fed infants, but serum or salivary measurements were not performed [40]. Human milk composition can also vary by maternal phenotype as females with type 2 diabetes and gestational diabetes have a greater human milk insulin concentration than females with normal glucose [41].

Although carbohydrate and oligosaccharide composition of human milk can be modified by maternal diet, lactose is consistently the primary carbohydrate measured in human milk [42]. As lactose is digested, galactose is also absorbed by SGLT1 in the intestines [43], which would increase 1,5-AG excretion. However, galactose administration to rats had a minimal impact on serum 1,5-AG concentrations [36]. Furthermore, human milk 1,5-AG concentration was not associated with infant body fat percentage or weight-for-age percentile at 1 or 6 mo of age [44]. Based on these findings, differences in salivary 1,5-AG concentrations in our cohort are unlikely due to dietary intake of 1,5-AG alone.

The current analysis used salivary 1,5-AG concentration as a biomarker for recent HEs. A strength of this study is that saliva 1,5-AG concentrations were measured using LC/MS. Enzymatic methods have reported that saliva galactose may interfere with saliva measurements, whereas LC/MS is able to distinguish the difference between molecules.

Salivary diagnostic or screening tests are of particular interest for research during infancy as saliva collection is noninvasive and does not require a skilled technician. These methods were validated for this cohort using established laboratory methods adopted from Jian et al. [19]. Saliva 1,5-AG has been previously validated as an alternative measure of HEs in adults [[19], [20], [21]]. Another strength of this work is that 1,5-AG and anthropometric data were collected prior to the introduction of solid foods, eliminating the impact of complementary foods as a confounding variable when considering feeding method. However, future research should evaluate 1,5-AG in the context of both the liquid diet and complementary feeding to assess the relationship between solid foods, human milk, and formula and HEs. More studies are needed to understand the mechanisms behind early infant feeding, glucose control, and the long-term impacts of hyperglycemia during infancy on body composition and growth trajectories to better inform future dietary guidance.

This study has limitations. Only 1 saliva sample taken at 5 mo of age was included in this analysis; more frequent sampling may provide a better indication of the relationship between mode of feeding and 1,5-AG concentration due to possible fluctuations in 1,5-AG values longitudinally. Although we did find differences in saliva 1,5-AG concentrations between infants consuming different types of formula, the sample sizes were relatively small, and the study should be repeated in a larger cohort aiming to compare formula types. The described sample of infants was from a mostly homogenous socioeconomic status. Although this allowed for a better comparison of outcomes, this work should be repeated in a larger, more diverse subject pool to increase the generalizability of the findings by including females with different maternal phenotypes.

In conclusion, we report that saliva 1,5-AG concentrations are associated with mode of feeding and infant weight gain between birth and age 5 mo. Human milk feeding was associated with higher 1,5-AG concentrations than formula feeding, suggesting that glycemic control differs by mode of feeding. As 1,5-AG was also associated with RWG, a relationship may exist between HEs and weight gain patterns in infants. The persistence of these findings into later childhood is an important area for future research. Efforts should aim to understand the mechanisms behind HEs in infancy as it relates to mode of feeding and growth trajectories.

## Author contributions

The authors’ responsibilities were as follows – DT, KM, SG, NFK, MT: designed the research plan, provided input on the project conception, and contributed to the study oversight; KM, MH, JK: worked with subjects to collect the data for this project; MA, KD: provided essential reagents and carried out the laboratory assays and the statistical analyses for the laboratory results; DT: performed the statistical analyses; KM, JK: provided input on the statistical approach for the study; DT, KM, SG, MT: were the primary authors involved with writing the manuscript; DT: had primary responsibility for final content; and all authors: read and approved the final manuscript.

## Data availability

The data used in this article will be made available upon request.

## Funding

Research reported in this publication was supported by the NIH-NIDDK grant number R01DK126710 (to MT). Supported by NIH/NCATS Colorado CTSA grant number UM1 TR004399. The content is solely the responsibility of the authors and does not necessarily represent the official views of the National Institutes of Health.

## Conflict of interest

Nancy F Krebs is an Editorial Board Member for *The American Journal of Clinical Nutrition* and played no role in the Journal’s evaluation of the manuscript. All other authors report no conflicts of interest.
